# ID 141 - Ferramenta para Subsidiar os Gestores na Decretação de Obsolescência de Dispositivos Médicos: aplicação a equipamentos

**DOI:** 10.5327/2237-9622.2025.v34s1.141

**Published:** 2025-11-25

**Authors:** Telma Vinhas Cardoso, Ana Lauren Martins de Oliveira

**Keywords:** dispositivo médico, equipamento médico-hospitalar, ciclo de vida, obsolescência

## Abstract

**Introdução:**

Muitos gestores têm dificuldades em decretar obsolescência de dispositivos médicos, principalmente quando se trata de equipamentos médicos (EM). Ao longo dos últimos anos, o Nats-Fatec/So recebeu solicitações dos setores de Engenharia Clínica (EC) de hospitais parceiros para justificar a retirada de equipamentos do seu parque tecnológico. Buscou-se, então, evoluir um modelo de avaliação de equipamentos na fase de grande utilização, adaptando-o para gerar pareceres quanto à fase de obsolescência. Gerar um aplicativo baseado em planilhas de dados vinculadas e habilitado a dar recomendações quanto à destinação dos EM em análise é o objetivo deste trabalho.

**Método:**

Seguiu-se uma metodologia de projetos, a partir da definição de indicadores de desempenho temporal, de qualidade e de custos, estabelecendo-se faixas para cada um deles. A seguir, foram estruturadas as telas de interface, com inserção de dados. As telas foram vinculadas, utilizando-se o MicroSoft Office Visual Basic for Applications. Foram adotadas estratégias de programação para gerar pareceres parciais e uma recomendação final.

**Resultados:**

Entre os indicadores da obsolescência técnico-funcional, destacam-se a idade do equipamento, o Tempo Médio Entre Falhas, o Tempo Médio para Reparo; o registro de Eventos Adversos/Alertas Sanitários e o Número de Ordens de Serviços do Equipamento. Na obsolescência contábil compara-se o custo das manutenções com o valor atual do equipamento e com o valor de um equipamento novo, estabelecendo faixas consideradas aceitáveis pelo usuário. Quanto à obsolescência tecnológica, avalia-se a complexidade do EM, verifica-se se há equipamento similar no mercado assim como a segurança, a eficácia e se o EM é utilizado pelos operadores.

**Conclusão:**

Considerando os indicadores definidos para cada dimensão da obsolescência, pode-se adotar pesos diferentes para aqueles indicadores considerados mais críticos de acordo com a filosofia da instituição. O usuário dispõe de relatórios parciais para cada tela com instruções para acompanhar as faixas predefinidas dos indicadores (que poderão ser mudadas de acordo com a realidade local) e comentários sobre o desempenho de cada um. Avaliando-se, então, as três dimensões de obsolescência, com pesos definidos, é gerado um relatório com a recomendação final de descarte imediato, a médio ou a longo prazos. Ao longo do seu desenvolvimento, o aplicativo foi avaliado pelos Setores de EC de dois grandes hospitais (São Paulo e Sorocaba) para gerar recomendações finais para decretação de obsolescência. Com as planilhas vinculadas, foi testado com a inserção de dados reais de EM, conduzindo a resultados coerentes. Existem aplicativos similares no mercado, inclusive com todas as etapas de gerenciamento do parque tecnológico. O aplicativo apresentado é exclusivo para a decretação de obsolescência, portanto, mais simples, e se baseia na utilização do aplicativo Microsoft Excel, de uso difundido. Naturalmente, admite melhorias principalmente quanto à segurança dos dados. Ainda assim, pode ser uma ferramenta de auxílio aos hospitais de pequeno e médio porte na gestão de seu parque tecnológico, dado que considera critérios técnicos, de segurança e de desempenho, o que pode permitir uma gestão mais estratégica e assertiva. Finalmente, há um grande aprendizado ao se fazer uma gestão contínua dos EM, pois isso pode influenciar os processos de compra de novos equipamentos, assim como sinalizar a necessidade da incorporação de novas tecnologias aos serviços de saúde.

